# Language proficiency and mental disorders among migrants: A systematic review

**DOI:** 10.1192/j.eurpsy.2021.2224

**Published:** 2021-07-28

**Authors:** C. Montemitro, G. D’Andrea, F. Cesa, G. Martinotti, M. Pettorruso, M. Di Giannantonio, R. Muratori, I. Tarricone

**Affiliations:** 1 Department of Neuroscience Imaging and Clinical Science, “G. d’Annunzio” University of Chieti, Chieti, Italy; 2 Department of Biomedical and NeuroMotorSciences (DIBINEM), Section of Psychiatry, University of Bologna, Bologna, Italy; 3 Department of Pharmacy, Pharmacology and Clinical Science, University of Hertfordshire, Herts, United Kingdom; 4 Department of Medical and Surgical Sciences (DIMEC), University of Bologna, Bologna, Italy; 5 Department of Mental Health, AUSL Bologna, Bologna, Italy

**Keywords:** language proficiency, mental disorders, migrants, migration

## Abstract

**Background:**

In this review, we aimed to evaluate the association between language proficiency (LP) and the prevalence and severity of mental disorders in migrants. Secondarily, we aimed to consider whether sociodemographic and migration-related factors may affect the correlation between LP and mental disorders.

**Methods:**

MEDLINE, PsycArticles, EMBASE, and PsycInfo were systematically searched in April 2020 to identify original studies reporting prevalence of psychiatric symptoms or disorders among migrants and taking into account linguistic factors.

**Results:**

The search of electronic databases initially yielded 1,944 citations. Of the 197 full texts assessed for eligibility, 41 studies were selected for inclusion in the systematic review. Thirty-five of the papers included reported a significant negative association between low LP and prevalence and/or severity of psychiatric symptoms or disorders, whereas only two records found the opposite relationship and four papers reported no association between them. Inadequate LP was consistently associated with several mental disorders in migrants, including psychotic, mood, anxiety, and post-traumatic stress disorders. Notably, all the four longitudinal studies that met inclusion criteria for this review reported a positive effect of LP acquisition over time on prevalence or symptom severity of mental disorders.

**Conclusions:**

Even though larger prospective studies are needed to better evaluate the relationship between LP and psychiatric disorders among migrants, we believe that the present findings could be inspiring for authorities to provide support and courses to improve migrants’ language proficiency upon arrival.

## Introduction

As defined by the World Health Organization (WHO), a migrant is any person moving from one area to another for varying periods of time [1]. According to the United Nations Department of Economic and Social Affairs, the number of international migrants increased from 153 to 272 million during the period from 1990 to 2019 [2]. Nowadays, migrants represent 3.5% of the world’s population, and it is estimated that 10.6% of them are refugees [2]. Moreover, hundreds of millions of people move within a country [3]. Migration should be considered as a complex process along which migrants may be exposed to several stressors, including stressful premigratory experiences, a traumatic act of migration, and postmigratory difficulties. The complex interplay between these factors, along with biological and psychological determinants, can lead to poor mental health [4]. For instance, in order to explain the higher prevalence of psychotic and schizophrenia spectrum disorders among migrants and ethnic minorities compared to native-born individuals, Morgan et al. [5,6] have proposed the existence of a socio-developmental pathway to psychosis. According to this model, early exposure to adversities which may occur prior, during, or after migration may interact with biological predisposition (e.g., genetic risk) and affect the neurobiological development creating an enduring proneness to psychosis. The onset of psychosis may then be detonated by the cumulative effect of further stressors, such as psychosocial adversities or substance abuse. Recent evidence from the field of neuroimaging supports the hypothesis that adverse social factors may increase the risk of psychosis in migrants via dysregulation of the dopamine neurotransmission system [7]. Acquisition of good language proficiency (LP) in the host country language is a key factor for the postmigration adaptation process and seems to be crucial for both psychosocial and economic wellness [8]. Specifically, according to a recent meta-analysis about mental health among minorities in the United States, LP was found to be negatively related to depression, anxiety, psychological distress, and negative affect [9].

LP relies on the ability of the interpretation of the linguistic code (words, sentences, etc.) as well as of the contextual knowledge of background and metalinguistic information—known as pragmatics, helping the recipient to understand the specific meaning of the speaker/sender [10]. Likewise, according to the socio-cognitive approach to second language acquisition, LP is more than the simple product of new knowledge acquisition [11]. Second language acquisition requires the interplay of cognitive, behavioral, and social skills, and occurs “in, for, and by virtue of integrated mind–body–world ecologies” [12]. Moreover, it has long been known that self-confidence, motivation, empathic skills, and several other affective factors may influence the second language acquisition process and act as an affective filter [13].

Many studies have explored the social determinants of LP, including, among others, age at migration, educational attainment, host language exposure, reason for migration, and social interactions [14–16].

In terms of mental disorders, LP may be considered a postmigration mediating factor [4,5]. In fact, poor LP may prevent interpersonal communication and transmission of emotional states, leading to self-isolation and alienation. An explanation could be that the neural processes underlying second language acquisition are involved in social cognition processes and, above all, theory of mind (ToM) [16].

Interestingly, severe psychiatric illnesses are often related to deficits in one or more aspects of social cognition. Specifically, emotion processing, ToM, and social relationship perception seem to be altered in psychotic spectrum disorders, major depressive disorder, and bipolar disorder [17],

In this light, migrants with a personal or family history of mental disorders may present impairment in social cognition and subsequent difficulties in tasks such as language learning. Shortcomings in LP could then be considered as the expression of a premigratory vulnerability to psychosis and other psychiatric disorders.

We aimed to review the studies analyzing the relationship between LP and (a) psychiatric disorder prevalence and/or severity among migrants and (b) psychiatric symptoms prevalence and/or severity among migrants. Secondarily, we aimed to consider whether sociodemographic and phase-specific migration factors may influence the relationship between LP and mental disorders.

## Methods

The Preferred Reporting Items for Systematic Reviews and Meta-Analyses (PRISMA) statement [18,19] and the recommendations of the Cochrane Collaboration [20] were followed for the systematic literature search.

### Study types

Eligible articles included all English language papers published in peer-reviewed journals from January 1990 to June 2021, reporting primary studies and data on mental disorders in first- or further-generation immigrants and investigating proficiency in the host language. References from each study were screened and reviewed. We limited the search to studies published after 1990 to ensure that all included studies rely on the same definition of “migrants,” as first defined by the United Nations General Assembly [21]. The last literature search was conducted on June 16, 2021.

### Search methods

MEDLINE, PsycArticles, EMBASE, and PsycInfo were systematically searched in April 2020 using controlled vocabulary and keywords to identify original studies reporting mental disorder prevalence among migrants and taking into account LP. Complete search algorithms are reported in Table S1 (see Supplementary Materials).

### Population and selection of studies

After performing the initial search, duplicates were identified and discarded. Titles and abstracts were screened and full texts were checked for eligibility in order to find potentially relevant reports. Studies were excluded if the full texts were unavailable even after contacting the corresponding authors. Three of the authors (G.D., C.M., and F.C.) performed the search and the initial data extraction independently, removing duplicates and all irrelevant articles after reading the specific abstract. The full texts of the remaining studies were independently assessed for eligibility by all authors. A consensus conference with the entire team took place in case of ambiguity.

In order to be included, the screened studies had to:

1. Examine a population of first- or further-generation adult migrants (18 years or older) as defined by the WHO [1]. Further definitions (e.g., on the basis of reasons for migration, legal and economic status) are beyond the scope of this paper. People born in the same country of residence from parents of different origin have been considered as further-generation immigrants and identified based on self-declaration.
2. Assess mental disorders using validated questionnaires, subscales, semi-structured interviews, or Diagnostical and Statistical Manual of Mental Disorders (DSM)/ International Classification of Diseases (ICD) diagnostic criteria.
3. Assess migrants’ LP.
4. Examine the relation between LP and mental disorders.
5. Have been published after 1990.

### Data extraction and analysis

The following information was extracted from the studies and reported in Table 1: (a) authors and year of publication; (b) country in which the study was conducted; (c) population studied; (d) migration history (reason for migration, length of stay, country of origin, destination country, and migrant generation); (e) study design; (f) psychometric measures; (g) language assessment instruments; (h) prevalence and/or severity of mental disorders; and (i) LP and correlation with mental disorders. A narrative synthesis was performed to analyze relevant literature.

**Table 1.** Included studies.

Reference	Country	Population target	Migration History	Design	Psychometric Measures	Language assessment	Main results	Language proficiency results	Rating
Furnham et al. [22]	UK	Chinese immigrants in the UK *N* = total 70 (43 FG 60; 27 SG) Age: 18–30 Gender = N/A Ethnicity = Chinese	Reason for migration: N/A Age of Migration: 61% ≥18 39% <18 Country of origin: Hong Kong Destination country: U.K Generation: 61% FG 39% SG	Cross-sectional study with control group	Langner-22 BDI Sociodemographic variables, social support, expectations	Self-reported LP	Langner-22: FG = 7.94 SG = 7.21 (*p* < 0.001) No significant differences on BDI between the two groups	Total sample: LP inversely correlated with psychological symptoms (*p* < 0.01) and MDD (*p* < 0.05) SG: LP inversely correlated with BDI (*p* < 0.05) but not with Lagnner-22 scores	3
Haasen et al. [23]	Germany	Migrants psychiatric inpatient clinic admitted from 1993 to 1995 *N* = 408 Age = 34.0 ± 11.6 Gender = 49.6% ♀ Ethnicity = Turkey 19.9%, Ex-Yugoslavia 13.7%, Europe 34.0%, Near East 13.7%, Far East 6.3%, Africa 6.6%, America 5.6%	Reason for migration: Asylum seeking/not specified Age at Migraton: 20.4 ± 9.7 Country of origin: See “Ethnicity” Destination country: Germany Generation: 100% FG	Cross-sectional study	ICD-10 (F1-F4) Sociodemographic and migration variables, course of illness, basic symptomatology, medication	Clinician report of “Language problems”	SCZ 38.7%, BD 3.7%, MDD 12.5%, SUD 11.5%, Mean Age at onset was 28.6 ± 10.1 85.2% onset of illness after migration	29.9% “Language problems” (59% Africans, 41% Near East, 36% ex-Yugoslavia, 29% East Europe, 20% other) SCZ subjects had more language problems (40%, *p* < 0.01)	1
Finch et al. [24]	United States	Adults of Mexican origin aged 18–59 living in California. 37.3% U.S. born 62.3% Mexico born *N* = 3.012 Age = 25.2% aged 18–24, 25.2% aged 25–34, 34.9% aged 35–44, 17.0% aged 45–59 Gender = 46.6% ♀ Ethnicity = Mexican	Reason for migration: N/A Proportion of life spent in the U.S: Max 63% Country of origin: Mexico Destination country: United States Generation: 63% FG	Cross-sectional study	CES-D Perceived discrimination scale Sociodemographic variables, acculturative stress including discrimination, legal status and social support	LP = 7-items 5-points Likert-type scale indicating relative usage of Spanish/English, with higher values representing greater usage of English Language conflict (modified HSI)	CES-D: 10.82 (mean)	LP = 2.58 (1–5) Higher LP predicted lower CES-D scores (*p* < 0.05). Higher levels of language acculturation were associated with more detrimental effects of perceived discrimination on depression (*p* < 0.05)	5
Ngo et al. [25]	United States	Vietnamese adults (aged ≥25) residing in the United States for at least 1 year and proficient in Vietnamese language *N* = 261 Age = 42.05 ± 12.40 Gender = 52% ♀ Ethnicity = Vietnamese	Reason for migration: Asylum seeking Age at Migration 100% ≥ 17 years Country of origin: Vietnam Destination country: United States Generation: 100% FG	Cross-sectional study	CES-D HTQ subscale for PTE Sociodemographic variables	LP = 6-items scale assessing the Vietnamese/English relative use in various social and intellectual situations. Higher scores refer to higher levels of acculturation	CES-D: 12.23 ± 8.12 PTE: 14.28 ± 4.58	LP: 14.28 ± 4.58 No correlation between LP and MDD. LP moderates the degree of negative impact of PTE on depression(*p* < 0.001) The impact of PTE on depression was greater for low LP (*p* < 0.01) than high LP (*p* < 0.01)	4
Miller et al. [26]	United States	Women from FSU residing in Chicago metropolitan area *N* = 200 Age = 56.82 ± 5.31 Gender = ♀ Ethnicity = Jewish 79.5%, Russian/Ukrainian/Other 20.5%	Reason for migration: Asylum seeking-other political reasons Age at Migration 100% > 40 years Country of origin: Former Soviet Union Destination country: U.S Generation: 100% first	Cross-sectional study (data from the Migration and Health Project study)	CES-D Sociodemographic variables, DI scale RS	LP and English Usage subscales from the Acculturation and Cultural Assimilation Scale LP = 3 items (understand, read and speak) rated on a 4-point Likert scale ranging from 1 = not at all to 4 = very well	CES-D: 28.28 ± 10.61 Age, English usage, DI scores and RS were significant contributors (*p* < 0.0001) to the variation in CES-D scores (33% of variance explained)	LP: 7.27 ± 2.02 English Usage: 1.79 ± 0.69 LP and English usage negatively correlated with CES-D	4
Halcon et al. (2004) [62]	United States	Somali and Oromo refugees aged 18–25 years *N* = 338 (participation rate 97.1%) Age = 20.9 ± 2.1 Gender = 38.8% ♀ Ethnicity: Somali 34.3%, Oromo 64.7%	Reason for migration: Asylum seeking Age at Migration: 14.9 ± 3.5 Country of origin: Somalia, Ethiopia Destination country: US Generation: 100% FG	Cross-sectional study	PCL-C (DSM-IV criteria) for PTSD Sociodemographic information, current life circumstances, life before coming to the United States, experienced trauma, violence or deprivation collected through a questionnaire of 585 response items	LP: Self-reported “speak English easily,” “read English easily,” and “problems learning English”	Results differed by ethnicity and gender PCL-C: 31.8 ± 13.0	54.4% speak English easily, 59.8% read English easily, 49.1% problems learning English Results differed by gender, with females reporting lower proficiency (*p* < 0.001) Higher LP = lower PTSD symptoms (*p* < 0.01)	6
Marshall et al. [27]	United States	Adult Cambodian immigrants residing in Long Beach, Calif, obtained through a 3-stage random sampling *N* = 586 (response rate 87% ➔ *N* = 490) Age = 52 ± 13.4 Gender = 61% ♀ (weighted) Ethnicity = Khmer (Cambodians)	Reason for migration: Asylum seeking Year of immigration: 1983 MOE 3.5% Country of origin: Cambodia Destination country: United States Generation: 100% FG	Cross-sectional study	CIDI Cambodian 17-HTQ and some items of Bosnian 46-HTQ SECV sociodemographic information	LP = Self-rated as following: “not at all,” “poor,” “fair,” or “good”	PTSD 62% MDD 51% comorbidity 42% Strong dose–response relationship with traumatic exposure	Low LP = higher rates of PTSD and MDD	6
Beiser et al. [28]	Canada	Southeast Asian refugee in Canada *N* = 647 Age = 41 range: 26–88 Gender = 42,7 ♀ Ethnicity = Vietnamese and Laotian 56,6%, Chinese 43.4%,	Reason for migration: Asylum seeking- Age at Migration: N/A Country of origin: Vietnam Destination country: Canada. Generation: 100% first	Cross-sectional study (data from the third survey of longitudinal study Psychiatry Refugee Resettlement Project)	DAM Ethnic identity scale Unemployment Sociodemographic variables: Age, gender, marital status, level of education, ethnicity	LP = self- rated on a 3-point scale of 1 = none, 2 = little, 3 = good. For analysis data were dichotomized as “none or little” versus “well”		LP: none or a little 67.4%, well 32.4%. Low LP associated with DAM scores (*p* < 0.001). Low VS high LP in low ethnic identity = higher DAM scores (*p* < 0.001)	5
Cordero et al. [29]	United States	Women enrolled in the WIC program *N* = 74 Gender = ♀ Ethnicity = Mainland-born Puerto Ricans (PRM) 44.6%, Island-born Puerto Ricans (PRI) 25.7%, Latinas 29.7%	Reason for migration: N/A Age at Migration: 16.4 ± 10.4 Length of stay (years): 10.7 ± 7.3 Country of origin: 70.3% Puerto Rico: 29.3% Colombia, Dominican Republic, Ecuador, Honduras, Mexico, and Peru Destination country: Generation: 53.7% FG 44.6% SG	Cross-sectional study	PrimeMD-PHQ (DSM-IV) Exposure to the mental-health care system, use of psychotropic medications Sociodemographic and acculturation variables (culture, language, traditional beliefs)	LP = 5-item Short Acculturation Scale. Responses were averaged to form a composite language score ranging from 1 (no English) to 5 (only English). Scores of 2.8 or greater indicated good English proficiency	PRI had the highest prevalence of anxiety attacks (*p* < 0.05), PRM and PRI had high prevalence of subthreshold or major depressive disorder (*p* < 0.05). Groups did not differ on medication use or mental health service utilization	LP = 2.6 ± 1.1 significantly lower for Latinas (*p* < 0.01) Lower LP = higher prevalence of anxiety attacks (*p* < 0.01)	5
Alegria et al. (2007) [66]	United States	Latino populations residing in the United States including first- (58.60%), second- (21.01%) and third-generation (20.39%) immigrants *N* = 2.554 Age = 49.01% aged 18–34, 30.07% aged 35–49, 13.38% aged 50–64, 7.55% aged ≥64 Gender = 48.50% ♀ Ethnicity = Cuban 22.59%, Puerto Rican 19.38%, Mexican 33.98%, other 24.05%	Reason for migration: N/A Age at Migration: <12: 12.22% 13–17: 10,95% 18–34: 28,73% ≥ 35: 6,47% (41.6% US Born) Country of origin: Cuba, Puerto Rico, Mexico, and Others Destination country: U.S Generation: 58.6% first 21.10% second 20.39% third or later	Cross-sectional study (data from the National Latino and Asian American Study, NLAAS)	WHO-CIDI (DSM-IV) Sociodemographic data, ethnicity and migration factors	LP = Self-rating of ability to speak, read, and write English. Response dichotomized as “excellent/good” or “fair/poor”	Lifetime psychiatric disorder prevalence: M 28.1% (SE = 2.1%), F 30.2% (SE = 1.6%) Prevalence rates for both lifetime and 12-months disorders were higher among third-generation respondents and varied across subethnic groups	LP good or excellent: 50.81% (SE = 2.53) High VS low LP: M + F: Higher risk for both lifetime and past-year risk of psychiatric disorders (*p* < 0.01) M: Higher risk of lifetime and past-year MDD (*p* < 0.05), lifetime SUD (<.01), F: Higher risk of lifetime AD (*p* < 0.05) and SUD (<.01),	5
Takeuchi et al. [30]	United States	Asian populations residing in the United States *N* = 2.095 Age = 41.33 ± 0.88 Gender = 52.55% ♀ Ethnicity = Chinese 28.69%, Filipino 21.59%, Vietnamese 12.93%, Other Asians 36.79%	Reason for migration: N/A Age at Migration: <12: 12.72% 13–17: 5.08% 18–34: 41.64% ≥ 35: 17.49% Country of origin: see Ethnicity Destination country: U.S Generation: 76.94% FG 13.68% SG 9.38% third or later	Cross-sectional study (data from the National Latino and Asian American Study, NLAAS)	CIDI (DSM-IV) Immigration-related variables	LP = Single question: “How well do you speak English?” Responses dichotomized into “excellent/good” or “fair/poor”	Any lifetime disorder 17.30%, 12-month any disorder 9.19% Psychiatric disorders: U.S.-born women > foreign-born women	Low LP: 33.81% Men with excellent/good LP had significantly lower rates of psychiatric disorders.	4
Birman et al. [31]	United States	Adult Vietnamese refugees resettled in the United States *N* = 212 Age = 48.8 ± 7.1 Gender = 48.6 ♀ Ethnicity = Vietnamese	Reason for migration: Asylum seeking Age at Migration: 48.84 ± 7.14 Country of origin: Vietnam Destination country: United States Generation: 100% first	Cross-sectional study	Hopkins symptoms checklist (HSCL-25) Nicassio’s Alienation Scale Trauma Events Scale form the Harvard Trauma Questionnaire Political detention Language, Identity, and Behavior scale Social Support Microsystems Scales adaptation of Fazel and Young’s Quality of Life Scale	LP = 9-items 4-point Likert-type (Language Acculturation subscale of the LIB). Respondents rated their ability to speak and understand the language in different situations as 1 = not at all to 4 = very well	Ex-political detainees: higher trauma (*p* < 0.001) but not anxiety, depression, alienation or less life satisfaction	LP = 2.16 ± .071 Higher LP was significantly correlated with reduced anxiety (*p* < 0.05)	5
Kang et al. [32]	United States	Korean immigrants aged ≥65 living in a community setting in Arizona (AZ) compared with Korean immigrants living in New York City (NYC) ethnic enclaves AZ sample: *N* = 120 Age = 71.2 ± 5.0 Gender = 55% ♀ NYC sample: *N* = 100 Age = 72.30 ± 6.10 Gender = 74% ♀ Ethnicity = Korean	Reason for migration: N/A Length of Stay (years): 25.6 ± 12.7 Country of origin: South Korea Destination country: U.S Generation: 100% first	Cross-sectional study with control group (data from *2000 Survey of the Asian American Elders in New York City*)	GDS-SF (DSM-IV) Sociodemographic variables, life and acculturative stressors, coping resources	LP = self-assessment on the ability to read, write, and speak in English, on a 4-point Likert-type scale ranging from 0 = not at all to 3 = very well (total score range 0–9)	Mild–severe depression: 38.1% AZ (vs. 24% NYC; *p* < 0.05) GDS: 5.7 ± 4.9 AZ (vs. NYC 7.80 ± 5.80; *p* < 0.05)	LP = 1.38 ± 0.81 AZ (vs. 2.20 ± 2.25 NYC; *p* < 0.001) In the AZ sample, lower LP associated with higher GDS-SF scores (*p* < 0.01)	5
Fenta et al. [33]	Canada	Adult Ethiopian immigrants residing in Toronto *N* = 342 Age = 35.3 ± 7.2 Gender = 40.6% ♀ Ethnicity = Ethiopian	Reason for migration: 45% Asylum seeking 55% N/A Age at migration: 22.9 ± 6.4 Country of origin: Ethiopia Destination country: Canada Generation 100% first	Cross-sectional study	CIDI PTSD scale DIS Sociodemographic variables, pre- and postmigration factors	LP = self-rated on a 4-point Likert scale ranging from 1 = poor to 4 = excellent for ability to speak, read and write in English with a range of 1–12	MDD 9.8%, AD 3.0%, PTSD 5.8%, Any disorder 14.0% 1 or more somatic symptoms 63.2%, 5 or more 12.9%.	LP = 9.82 ± 2.2 Higher LP = less somatic symptoms, bivariate (*p* = 0.001) and multivariate linear regression analysis (*p* < 0.001)	4
Leong et al. [34]	United States	Latino and Asian populations residing in the United States Latino immigrants 57.1%, U.S.-born 42.9% Asian immigrants 76.1%, U.S.-born 23.7% *N* = 4.649 Age = Latinos 38.02 ± 15.03, Asians 41.34 ± 15.57 Gender = 51.5% Latino ♀, 52.5% Asian ♀ Ethnicity = Latinos 54.9%, Asians 45.1%	Reason for migration: N/A Length of stay (years): ≤10 years Country of origin: see “Ethnicity” Destination country: U.S: Generation: N/A	Cross-sectional study (data from the National Latino and Asian American Study, NLAAS)	WHO-CIDI Social networking, Ethnic identity, Discrimination, Acculturative stress, Family conflict, SES	Variable based on a combination of NLP and HLP. 3 items (speak, read, and write), rated on a 4-point scale ranging from 1 (poor) to 4 (excellent), and items were averaged to compute the scale scores. Participants scoring “high” on both NLP and HLP were coded as bilingual, those scoring low on HLP were coded as LEP	Immigrants reported higher levels of ethnic identity, family cohesion, NLP and lower HLP than their U.S.-born counterparts (*p* < 0.001). Social networking was a protective factor, while discrimination, acculturative stress, and family conflict were risk factors for poor mental health	Immigrants had significantly lower ELP than U.S.-born (*p* < 0.001) Bilingualism played a protective role against MDD among Asian immigrants (*p* < 0.05) No significant relation was found for Latinos	4
Bernstein et al. [35]	United States	Korean immigrants in NYC *N* = 304 Age = 46.7 ± 14.3 Gender = 56.6% ♀ Ethnicity = Korean	Reason for migration: N/A Age at Migration: <17 = 10.2% 18–34 = 51% ≥35 = 38.8% Country of origin: South Korea Destination country: United States Generation: 100% first	Cross-sectional study	CES-D Discrimination Scale Acculturative Stress Scale	LP = 3-items (“How well do you speak English?” “How well do you write English?” and “How well do you read English?”) 4-point Likert-type scale with a score ranging from 3 to 12 and higher scores indicating greater proficiency. Scores were dichotomized as “poor/fair” or “good/excellent” for analysis	CES-D: 11.59 ± 9.7, 13.2% of the sample scoring higher than cut-off (21). Self-reported exposure to discrimination was related to higher depressive symptoms in hierarchical multiple regression analysis (*p* < 0.01)	Poor/fair in speaking = 78.3%, reading = 76.6%, writing = 80.6% Low LP associated with MDD (*p* < 0.01) even if controlled for sociodemographic factors, years in the United States, and acculturative stress (*p* < 0.05)	6
Kim et al. [36]	Korea	Female immigrants residing in Korea after international marriage *N* = 466 Age = 29.72 ± 7.11 Gender = ♀ Ethnicity = Asians (Vietnam, China, Philippines, Other)	Reason for migration: Work? Length of stay (years): 4.15 ± 2.69 Country of origin: 89% China, 35% Japan, 35% Philippines, 31% Thailand and Mongolia Destination country: Korea Generation 100% first	Cross-sectional study	Depression assessed with CES-D Korean language ability	LP = 4-subscales 5-point Likert-type instrument assessing speaking, listening, reading, and writing skills. The score ranges from 4 to 20	CES-D: 14.10 ± 9.07, with 39.9% above the cut-off (16) CES-D in MDD patients (≥16): 23.06 ± 6.26 MDD: Chinese (OR = 3.506, 95%CI = 1.462–8.411, *p* = 0.005) and in participants from other countries (OR = 2.539, 95%CI = 1.070–6.026, *p* = 0.035)	LP = 12.65 (mean) (MDD = noMDD). Higher LP protected against depression (OR = 0.561, 95% CI = 0.325–0.983, *p* < 0.05)	6
Kim et al. [37]	Canada	Elderly Korean immigrants living in Toronto *N* = 148 Age = 74.01 ± 6.24 Gender = 61.5% ♀ Ethnicity = Asians	Reason for migration: N/A Length of stay (years): 21.01 ± 11.01 Country of origin: South Korea Destination country: Canada Generation: 100% first	Cross-sectional study	CES-D Demographic acculturation social variables	LP rated by interviewer as: “not at all” (0), “not much” (1), “some” (2), “very well” (3)	CES-D: 12.39 ± 9.78, Women > Men (*p* < 0.05)	LP = Not at all 23.0%, Not much 25.7%, Some 38.5%, Very well 12.8% Low LP did not predict depression	4
Kang et al. [38]	United States	Chinese immigrants aged ≥65 living in a community setting *N* = 120 Age = 75.6 ± 6.9 Gender = 57.8% ♀ Ethnicity = Chinese	Reason for migration: N/A Length of stay (years): <18 Country of origin: China Destination country: United States Generation: N/A	Cross-sectional study	GDS-SF (DSM-IV) Sociodemographic variables, life and acculturative stressors, coping resources	LP = self-assessment on the ability to read, write, and speak in English, on a 4-point Likert-type scale ranging from 0 = not at all to 3 = very well (total score range 0–9)	GDS-SF: 5.7 ± 4.9, 14.2% mild/moderate to severe depression	LP = 4.05 ± 2.85 Low LP significantly associated with higher GDS-SF scores (*p* < 0.05)	6
Nicholson et al. [39]	United States	Immigrants from Former Soviet Union residing in Chicago Metropolitan Area *N* = 137 Age = F 61.0 ± 8.1, M 64.2 ± 8.1 Gender = 51.4% ♀ Ethnicity = Russian	Reason for migration: Asylum seeking-Other political reasons Length of stay (years): 6.2 ± 2.1 Country of origin: Former Soviet Union Destination country: United States Generation: 100% first	Cross-sectional study	Depression assessed with CES-D Language and Environmental Mastery CPSS Alienation Scale Salivary Cortisol	LP = 3 items 4-point Likert-type instrument assessing understanding, reading and speaking in English, ranged from 1 = not at all to 4 = very well	Higher environmental mastery associated with lower CES-D	No significant association between LP and CES-D Language mastery decreased alienation in women	4
Kang et al. [40]	United States	Korean immigrants aged ≥65 living in the community and being cognitively intact *N* = 120 Age = 75.7 ± 7.1 Gender = 69.2% ♀ Ethnicity = Korean	Reason for migration: N/A Length of stay (years): 24.9 ± 8.21 Country of origin: South Korea Destination country: U.S: Generation: N/A	Cross-sectional study	GDS-SF (DSM-IV) Sociodemographic variables Life and acculturative stressors Coping resources	LP = self-assessment on the ability to read, write, and speak in English, on a 4-point Likert-type scale ranging from 0 = not at all to 3 = very well (total score range 0–9)	GDS-SF = 4.77 ± 2.85 37.5% mild/moderate to severe depression	LP = 2.09 ± 2.39 Low LP significantly associated with high GDS-SF scores (*p* < 0.01)	4
Kim et al. [41]	Korea	Female immigrants residing in Korea after international marriage *N* = 223 Age = 29.4 ± 7.37 Gender = ♀ Ethnicity = Asians (China, Vietnam, Philippines, and Japan)	Reason for migration Wedding Length of stay (years): <1 10.59%; 1–3 9.64%; 3–5 11%; >5 years 10.45% Country of origin: 35.2% China, 29.7% Vietnam, 10.5%, Philippines, 5% Japan Destination country: Korea Generation 100% FG	Cross-sectional study	CES-D VIA SAFE	LP = 4-items 10-point VAS scale assessing speaking, listening, reading, and writing in Korean (0 = very poor, 10 = very fluent)	CES-D = 10.31 ± 5.88 SAFE = 39.95 ± 7.45	LP = 19.39 ± 8.34 No significant association between LP and depression	5
Kim et al. [42]	Korea	Female immigrants residing in Korea after international marriage *N* = 173 Age = 28.6 ± 6.2 Gender = ♀ Ethnicity = Asians (China, Japan, Philippines, Vietnam, Mongol, and Others)	Reason for migration: Wedding Length of stay (years): 0–2 17.3%; 2–4 33.5%; 4–6 25.4%; 6–8 13.9%; ≤8 9.8% Country of origin: 49.1%Vietnam, 26.5% China, 16.2% Others, 2.9% Japan, 2.9% Philippines, 2.3% Mongol Destination country: Korea Generation 100% FG	Cross-sectional study	CES-D ASS	Korean Language Literacy Scale (KLLS, created ad hoc), a 4-items 5-point (1 = poor, 5 = excellent) likert scale assessing speaking, writing, reading, and understanding Korean. The score ranges from 4 to 20	CES-D = 12.14 ± 9.25 9.6% above the cut-off (21) Acculturative stress (*p* < 0.001) and life satisfaction (*p* = 0.003) were significantly associated with CES-D score	KLLS = 10.3 ± 3.6 LP was not significantly correlated with depression	5
Nickerson et al. [43]	Australia	Adults from Mandaean community residing in Sidney *N* = 367 (response rate 86% ➔ *N* = 248) Age = 38.31 ± 14.53 Gender = 52% ♀ Ethnicity = Mandeans	Reason for migration: Asylum seeking-other political reason Length of stay (years): 4.31 ± 4.25 Country of origin: See Ethnicity Destination country: Australia Generation 100% FG	Cross-sectional study	HTQ ICG HSCL-D Postmigration living difficulties,	LP assessed with ISLPRS, yielding a continuous score ranging from 0 (no ability) to 7 (communicating like a native speaker)	Four classes: PTSD/PGD 16%, PTSD 25%, PGD 16%, resilient 43%. Classes 1, 2, and 3 had significantly higher levels of depression.	LP did not predict any symptom	4
Rask et al. [44]	Finland	Russian, Somali and Kurdish immigrants residing in Finland. Finnish natives (n = 956) from the national sample of the health 2011 Survey *N* = 2.316 Age = 18–64 years (mean n/a) Gender = 55.96% ♀ Ethnicity = Kurdish, Russian, Somali	Reason for migration: Asylum seeking- not specified Age at migration 70% ≥ 18 30% < 18 Country of origin: 34% Russia, 28% Somalia, 38% Kurdistan Destination country: Finland Generation: 100% FG	Cross-sectional study (data from the Finnish Migrant Health and Wellbeing Study (Maamu) conducted in 2010–12)	HSCL-25 SCL-90 Sociodemographic variables	LP = self-rated as following: “not at all,” “poor,” “good,” or “fair” “not at all” and “poor” were grouped together as “low”	MDD and AD: Kurdish 23.0%, Russian 18.0%, Somali 8.5% Somatization: Kurdish 28.9%, Russian 14.8%, Somali 12.9% Higher HSCL-25 in Russian (*p* = 0.001) and Kurdish (*p* < 0.001) women	Low LP predicted significantly higher HSCL-25 scores in Russian (OR 3.46, CI 1.50–7.98) and Kurdish (OR 2.15, CI 1.09–4.21), but not Somali women	4
Morawa et al. [45]	Germany	Individuals of Turkish origin residing in Essen. Foreign-born 82.7% *N* = 335 Age = 41.6 ± 11.3 Gender = 63% ♀ Ethnicity = Turks	Reason for migration: N/A Age at migration 20.2 ± 8.2 Country of origin: Turkey Destination country: Germany Generation: 82.7% FG 17.3% SG	Cross-sectional study	PHQ-9 PHQ-15 Sociodemographic and migration-specific variables Physical illnesses	LP = self-rated as following: “German as mother tongue,” “Very good,” “Good,” “Moderate,” “Little,” and “Bad”	PHQ-15 ≥ 15 = 24.2% PHQ-9 ≥ 15 = 16.7% comorbidity 53.1%	German as mother tongue 8.5%, Very good 15.2%, Good 22.4%, Moderate 24.8%, Little 15.2%, Bad 3.6%, No data 10.3% Better LP was significantly associated with lower PHQ-15	4
Schweitzer et al. [46]	Australia	Refugee women recently resettled in Australia *N* = 104 Age = 32.5 ± 11.6 Gender = ♀ Ethnicity = African 78.9%, Asian 21.1%	Reason for migration: Asylum seeking Length of stay (years) <0.5 Country of origin: 78.9% Africa (Eritrea, Democratic Republic of Congo, Ethiopia, Sudan, South Sudan, Rwanda, Burundi, and Kenya), 21.1% Asia (Afghanistan, Iran, Iraq, Syria, Myanmar and Thailand) Destination country: Australia Generation: 100% FG	Cross-sectional study	HTQ HSCL-37 Demographic questionnaire Postmigration Living Difficulties Checklist	LP = self-rated as following: “no skills,” “great difficulty,” “some difficulty,” or “fluent”	PTSD 20% AD 29% MDD 41%, Somatization 42%	“No skills” or “great difficulty” = 61.5% Greater LP predicted higher anxiety symptoms (*p* < 0.05)	4
An et al. [47]	South Korea	Chinese immigrants in South Korea *N* = 128 Age = Mode (40–49) 40.5% Gender = 85.2% ♀ Ethnicity = Chinese	Reason for migration: N/A Length of stay (years): <5: 14.1%, 5–10: 29.7%, 10–15: 23.4%, 15–20: 14.1%, >20: 11.7% Country of origin: China Destination country: South-Korea Generation 100% FG	Cross-sectional study	Health Literacy Scale BDI Immigrant stress Sociodemographic variables	LP = Self-assessed Korean (speaking, listening, reading, writing) reported as follow: “poor,” “average,” and “good”	MDD 20%	Low LP = 6.3% Poor LP associated with lower Health literacy (*p* < 0.001) and higher Immigrant stress (*p* < 0.001) LP did not influence depression	4
Kartal et al. [48]	Australia Austria	Individuals >18 years old exposed to war in Bosnia during 1992–95 and residing in Australia or Austria *N* = 138 Age = 40.20 ± 14.91 Gender = 45% ♀ Ethnicity = Bosniak 80%, Mixed 4%, Croatian Bosnian 1%, Serbian Bosnian 1%, not declared 15%	Reason for migration: Asylum seeking Length of stay (years): 18 ± N/A Country of origin: Bosnia Destination country: Australia, Austria Generation: 100% FG	Cross-sectional study	Trauma exposure PDS (DSM-IV) DASS-21	LP = LIB (Language acquisition subscale)	Reported trauma = ≥ 1: 82% ≥3: 70% PTSD (mild to severe): 34.1% MDD (mild to severe): 30% AD (mild to severe): 20%	Trauma exposure positively associated with PTSD, MDD and AD, and negatively associated with LP LP negatively associated with PTSD (−.411), MDD (−.296) and AD (−.359) LP mediated the relation between trauma exposure and PTSD (*p* = 0.028) and AD (*p* = 0.026)	6
Carl et al. [49]	United States	Puerto Ricans migrated in the United States after Hurricane Maria versus Puerto Ricans residents (control) *N* = 107 Age = 35.3 ± 1 Gender = 64.2% ♀ Ethnicity = Hispanic/Latino 92%, Others 8%	Reason for migration: Other political reasons Length of stay (years): ≥ 0.25 Country of origin: Puerto Rico Destination country: United States Generation: 100%FG	Cross- sectional study with control group	K6 GAD-7 PCL-5 PHQ-9 Sociodemographic variables	Preferred language for questionnaire completion (English vs. Spanish) Responders self-assessment of their ability to speak and understand English on a 2 item 4 points Likert type scale	Prevalence of severe mental disorders: migrants = controls	Prevalence of severe mental distress (K6 > 13): Spanish-preferring migrants (30.4%) > English-preferring migrants (0%) (*p* = 0.021) Controls (9.6%)	4
Jongsma et al [50]	Multicentric (6 countries)	FEP migrants versus native HR *N* = 1130 Age = 29 ± N/A Gender = 38.3%% ♀ Ethnicity: White majority 634 (58.3%), Black 168 (15.4%), Mixed 107 (9.8%), Asian 33 (3.1%), North African 45 (4.1%), Other 29 (2.8%) White other, 72 (6.8%)	Reason for migration: N/A Age at migration: N/A Country of origin: See Ethnicity Destination country: UK, The Netherlands, France, Spain, Italy, and Brazil Generation 53% FG 47% SG	Case–Control study (data from EU-GEI study database)	ICD Major Experiences of Discrimination questionnaire	Linguistic distance = language distance (0–3) + fluency in the majority language (0–10). No linguistic distance (language distance = 0, fluency = 10) or some linguistic distance (language distance ⩾1 and/or fluency ⩽9).	Higher odds of psychosis in ethnic minority background, attenuated when corrected for social disadvantage and linguistic distance Linguistic distance = stronger effects for FG Social disadvantage = stronger effects for SG	Cases VS controls = greater linguistic distance (*p* < 0.01) Linguistic distance = increased odds of psychosis (OR 1.89, CI 1.31–2.73, *p* < 0.05)	5
Beiser et al. [51]	Canada	Asian refugees resettled refugees in Canada. Assessment: 1981 (t1), 1983 (t2), and 1991 (t3) *N* = 608 Age = 41 (range 26–88) Gender = 43% ♀ Ethnicity = Asian (Chinese, Laotioan, Vietnamese)	Reason for migration: Asylum seeking Length of stay (years): ≥10 Country of origin: See Ethnicity Destination country: Canada Generation: 100% FG	Prospective cohort study	RRP symptom inventory (items from CES-D, SHS, DIS) Sociodemographic variables (Age, gender, education, employment)	LP = self-rated on a 3-point scale of “none” (1), “little” (2), or “good” (3)	MDD decreased over time (*p* < 0.001): 6.48% (t1) ➔ 4.37% (t2) ➔ 2.27% (t3). MDD at t1 correlated with MDD at t2 (*p* < 0.01) and at t3 (*p* < 0.01)	Good LP: 17.4% (t1) ➔ 25.3% (t2) ➔ 32.4% (t3) None LP: 16.3% (t1) ➔ 8.4% (t2) ➔ 7.7% (t3) Good LP at T1 associated with education, age and sex LP at T3: negative related with MDD	5
Sӧndergaard et al. (2004) [63]	Sweden	Recently resettled refugees from Iraq in Stockholm, who went through language training. Assessment: at baseline and every 3 months (4 T) *N* = 48 Age = 36 (range 20–48) Gender = 33% ♀ Ethnicity = Iraqi (Arabic- or Sorani-speaking)	Reason for migration Asylum seeking Age at migration N/A Country of origin: Iraq Destination country: Sweden Generation: 100% first	Prospective cohort study	PTSD IES-22 HSCL-25 DES Demographics	LP = number of training levels passed. Levels were 0–5: 4 is for access to work market, 5 is for access to higher education. Level attained at the final follow-up (9 m) corrected for (date of language data - date of health data)	PTSD: 39.6% MDD strongly associated with PTSD (*p* = 0.001)	PTSD = worse LP (*p* = 0.08) MDD unrelated with LP LP negatively associated with PTSD symptoms (*p* = 0.004) and HSCL-25 (*p* = 0.063)	4
Arevalo et al. (2015) [61]	United States	Puerto Ricans residing in the United States Assessment: baseline +2.2 years FU *N* = 1.205 Age = 57 ± 7.6 Gender = 71% ♀ Ethnicity = Puerto Ricans	Reason for migration: Work-family reunion-health-others Length of stay (years) <15: 9%, 15–24: 12%, 25–34: 22%, >35: 58% Country of origin: Puerto Rico Destination country: United States Generation N/A	Prospective cohort study (data from population-based prospective cohort study Boston Puerto Rican Health Study)	CES-D Neighborhood Ethnic Density and poverty level Migratory factors	LP = Acculturation Scale for Hispanics (ASH) modified for Puerto Rican population; scores range from 0–100, with higher scores indicating greater use of English vs. Spanish.	CES-D > 16: 60% (T1, T2) CES-D: 20.1 ± 13.1 (T1) ➔ 18.1 ± 12.5 (T2) (*p* < 0.001)	ASH categorized in quartiles Higher LP associated with lower MDD at FU (*p* < 0.05) High ethnic density neighborhoods: lower MDD in ASH Q2 (*p* < 0.05)	6
Kindermann et al. [52]	Germany	Asylum seekers recruited in a psychosocial outpatient clinic. Assessment: baseline (T1), 3 to 5 months (T2) *N* = 84 Age = 32 ± 8.7 Gender = 39.3% ♀ Ethnicity = East Europe 15.5%, Asia 52.4%, Africa 32.1%	Reason for migration Asylum seeking Age at migration: N/A Country of origin: See ethnicity Destination country: Germany Generation N/A	Prospective cohort study	PHQ-2 GAD-2 PHQ-PD PC-PTSD-5 ERQ-10 SOC-9 L Sociodemographic and cultural background variables	LP = Participants defined themselves as proficient or not proficient in English/German	PHQ-2: 4.3 ± 1.8 ➔ 3.4 ± 1.6 (ns) GAD-2: 4.2 ± 1.9 ➔ 3.9 ± 1.9 (ns) PHQ-PD: 3.3 ± 2.1 ➔ 2.6 ± 2.3 (ns) PC-PTSD-5: 3.1 ± 1.8 ➔ 3.4 ± 1.6 (ns)	High LP = 34.5% (n = 29) LP predicted reduction of MDD, AD and PTSD at T2 (*p* < 0.01)	4
Chen et al. [53]	United States	Chinese immigrant mothers (each participant had 1–4 children) *N* = 257 Age = 37.9 ± 5.9 Gender = 100% ♀ Ethnicity = Chinese (mainland China +2 Taiwan +1 Hong Kong)	Reason for migration NA Age at migration: 28.5 ± 6.5 Country of origin: China Destination country: USA Generation 100% FG	Cross-sectional study	CADS-9 ISEL-12 Sociodemographic and economic variables Annual income Shift in Subjective Social Status (SSS)	LP = self-rated on a continuous scale from “extremely poor” (1) to “very good” (5) in English	CADS-9: 4.95 ± 3.92 ISEL-12: 36.62 ± 6.23	No significant correlation between LP and depressive symptoms (*r* = −.08, *p* > 0.05) Mediation analysis: LP ➔ income, shifts in SSS, and interpersonal support ➔ depressive symptoms	3
Hamrah et al. [54]	Australia	Afghan refugees in Tasmania *N* = 66 Age = 18–79 years Gender = 24.6% ♀ Ethnicity = Afghan Hazaras	Reason for migration Asylum seeking Age at migration: N/A Country of origin: Afghanistan Destination country: Tasmania, Australia Generation N/A Length of Stay in NE: 27 (SE: 0.14) months	Cross-sectional survey	IES-R Postmigration living difficulties scale (PMLD)	LP reported as communication difficulties, as assessed by the PMLD	PTSD symptoms were observed in 32 subjects (49.5%)	Communication difficulties have been reported in PTSD more than in non-PTSD subjects (90.6% vs. 38.2%, *p* < 0.001) Communication difficulties were strongly associated with PTSD in different multivariate models	3
Hamrah et al. [55]	Australia	Afghan refugees in Tasmania *N* = 66 Age = 18–79 years Gender = 25.7% ♀ Ethnicity = Afghan Hazaras (Population already described in Hamrah et al., 2020)	Reason for migration Asylum seeking Age at migration: N/A Country of origin: Afghanistan Destination country: Tasmania, Australia Generation N/A Length of Stay in NE: 27 (SE: 0.14) months	Cross-sectional survey	HSCL-25 PMLD: 25 items assessing living difficulties likely to be experienced by resettled refugees, such as isolation, communication difficulties, etc.	LP reported as communication difficulties, as assessed by the PMLD	Depressive symptoms were observed in 14 subjects (21%)	Participants with depressive symptoms showed higher levels of communication difficulties (*p* = 0.053) No association reported between LP and depressive symptoms in a multivariate analysis	3
Hamwi et al. [56]	Portugal	Adult migrant and native women who had a live birth in one of the maternity units between April 2017 and March 2019. *N* = 1475 migrants +1,415 native women Age = 18–79 years Gender = 100% ♀	Reason for migration N/A Age at migration: N/A Country of origin: Brazil, Portuguese speaking African Countries, other African Countries, Europe, Asia, America Destination country: Portugal Generation N/A Length of Stay: Reported only by categories	Prospective cohort study	EPDS	LP categorized into native, full, intermediate, or limited LP in Portuguese Migrants were categorized based on self-report in 4 areas (understanding, speaking, reading, and writing) on a scale from 0 (no proficiency), to 3 (full proficiency) -full: 874 (59.3%), -intermediate: 412 (27.9%) -limited: 189 (12.8%)	EPDS scores >10 were observed in 12.4% of migrant and 7.2% of native women (*p* < 0.001)	LP categories showed significant differences in EPDS (*p* < 0.001) - natives 7.2% - full LP 11.3% - intermediate 12.6% - limited 18% Negative relationship between EPDS values and LP. Full, intermediate, and limited LP were associated with higher risk of postpartum depression compared to native (p’s < 0.001) The effect of LP was stronger among recent migrants.	5
Ventriglio et al. [57]	Italy	Migrant patients who have been hospitalized at the Psychiatric Unit from 2004 to 2018 *N* = 243 Age = 34.48 + 10.26 Gender = 42.39% ♀ Ethnicity 18.4% Africa, 12.3% America, 10.3% Asia, 48.6% Europe	Reason for migration N/A Age at migration: N/A Country of origin: See Ethnicity Destination country: Italy Generation N/A Length of Stay: N/A	Retrospective cohort study	DSM-IV-TR	LP evaluated as inadequate if clinicians needed mediator/translator	DSM-IV TR diagnoses (%): Psychoses: 30.8 MDD: 25.1 substance use:15.6 Bipolar Disorder: 11.9 Anxiety: 6.58 Suicidal attempt: 18.5 First-episode: 42.3% 90.9% of patients reported a stressful event before hospitalization	First- episode of mental illness was significantly associated with lower Italian LP (all *p* < 0.0009).	2
Wu et al. [58]	Australia	Humanitarian migrants arrived in Australia W1 (October 2013 to March 2014) *N* = 2,399 Gender = 45.5% ♀ Age: M = 35.7 + 13.8, F = 35.2 + 14 W2 (October 2014 to March 2015) *N* = 2009 Gender = 44.6% ♀ Age: M = 36.7 + 13.8, F = 36.4 + 14.1 W3(October 2015 to March 2016) *N* = 1,894 Gender = 46.8% ♀ Age: M = 38.5 + 14.2, F = 37.8 + 14.2 W4 (October 2016 to February 2017) *N* = 1,929 Gender = 46.5% ♀ Age: M = 39.4 + 14.1, F = 38.5 + 14.3	Reason for migration N/A Age at migration: N/A Country of origin: N/A Destination country: Australia Generation N/A Length of Stay: N/A	Longitudinal study	PTSD-8 K6 Premigration factors: n. of traumatic events experienced Postmigration factors: economic stressors, English language barriers, family conflicts in Australia, loneliness, discrimination, concerns about their family in Australia and adjustment to life	LP indirectly assessed as presence or absence of English Language Barriers, as reported by subjects during the interview	PTSD prevalence decreased across Waves and was higher in women than in men [women: 36.6% W1, 30.5% W2, 32.8% W3, 22.8% W4; men: 28.1% W1, 22.6% W2, 27.9% W3, 18.5% W4] HR-SMI prevalence decreased over time and was higher in women than in men [women: 20.1% W1, 16.1%W2, 19.7% W3, 15.3% W4; men: 12.5% W1, 11.2% W2, 13.9% W3, 10.0% W4	There were no significant relationships between LP and female refugee’s PTSD and HR-SMI. A positive relationship was only found in the W4 male refugees, were Language barriers predicted higher PTSD scores (*p* = 0.012)	5

Psychometric Measures are extensively reported in Table S2.

Abbreviations: AD, anxiety disorders; ADJ, adjustment disorder; ASH, Acculturation Scale for Hispanics; ASS, Acculturative Stress Scale; BD, bipolar disorder; BDI, Beck Depression Inventory; CCM, collaborative care management; CES-D, Center for Epidemiologic Studies Depression Scale; CI, confidence interval; CIDI, Composite International Diagnostic Interview; CPSS, Cohen’s Perceived Stress Scale; DAM, Depressive Affect Measure Scale; DES, Depression Anxiety and Stress Scale;  DSM, Diagnostical and Statistical Manual of Mental Disorders; FEP, first-episode psychosis; FG, first generation; FU, follow-up; GDS-SF, Geriatric Depression Scale Short Form; HR-SMI, high risk of severe mental illness; HTQ, Harvard Trauma Questionnaire; ICD, International Classification of Diseases; ICG, Inventory of Complicated Grief; IES, Impact of Events Scale; ISLPRS, International Second Language Proficiency Rating; KLLS, Korean Language Literacy Scale; LIB, Language, Identity, Behavioral Acculturation Scale; LP, language proficiency; MDD, major depressive disorder; *N*, number; NLAAS, National Latino and Asian American Study; OR, odds ratio; PCL-C, Post-Traumatic Stress Disorder Checklist-Civilian Version; PD, personality disorders; PGD, prolonged grief disorder; PHQ, Patient Health Questionnaire; PPV, permanente protection visas; PTE, Premigration Traumatic Experiences; PTSD, post-traumatic stress disorder; PV, political violence; RRP, Refugee Resettlment Project; RS, resilience; SAFE, Social, Attitudinal, Familial, and Environmental Acculturative Stress Scale; SCZ, schizophrenia spectrum disorders; SE, standard error; SECV, Survey of Exposure to Community Violence; SES, Socio-Economic Status; SG, second generation; SHS, Senegal Health Scale; SUD, substance use disorder; TPV, temporary protection visas; WIC, The Special Supplemental Nutrition Program for Women, Infants, and Children.

### Quality rating

The quality of the studies was gauged considering the seven items of the quality assessment checklist for observational studies (QATSO Score) [59] adapted to our search. In particular, the checklist evaluates: (a) sample size and source; (b) use of validated tools for psychiatric measures; (c) use of validated tools for LP assessment; (d) reports of the response rate; (e) reports of migration history data (reason for migration, length of stay, country of origin, destination country, and migrant generation); (f) checking for confounding factors (e.g., stratification/matching/restriction/adjustment); and (g) privacy and ethical aspects considered. The QATSO score is reported in Table 1.

## Results

**Figure 1.**
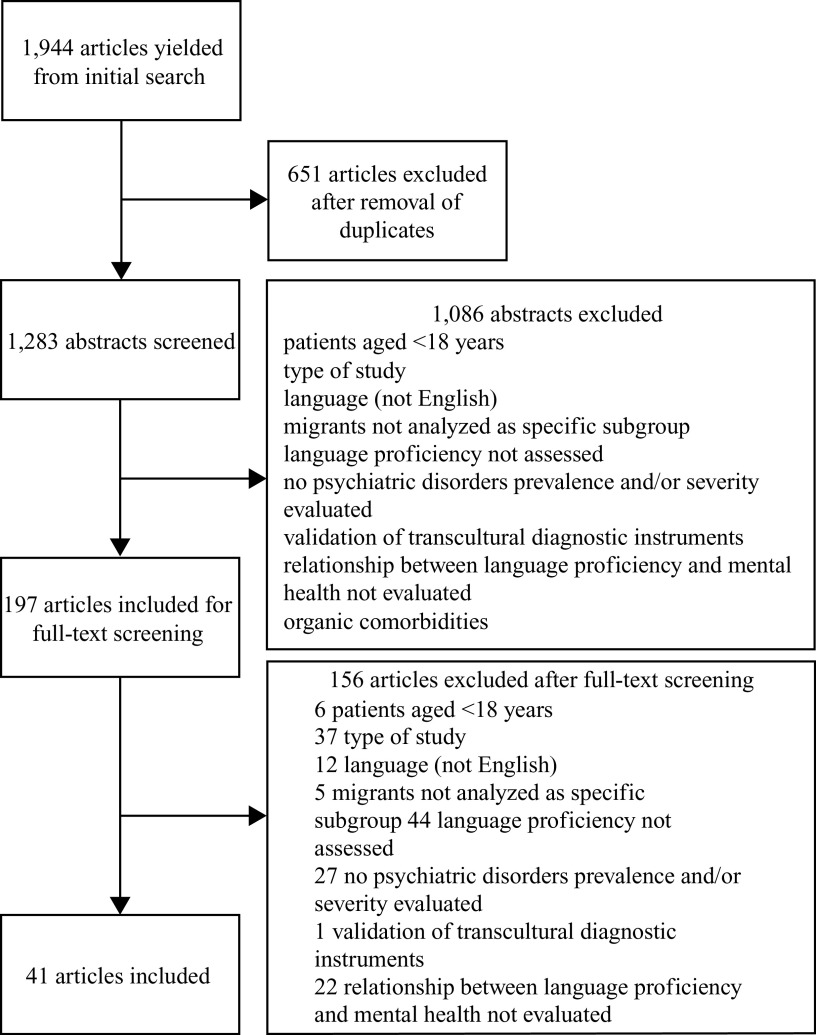
Preferred Reporting Items for Systematic Reviews and Meta-Analyses (PRISMA) flowchart.

Searching of electronic databases initially yielded 1,944 citations, as reported in the PRISMA flowchart (Figure 1). After removing duplicates, 1,283 citations remained. Of these, 1,086 citations were excluded after reading the abstract, as they were reviews, meta-analyses, commentaries, letters to the editor, dissertations, books or book chapters, or non-English language works, or because they did not meet the inclusion criteria. Of the 197 full texts assessed for eligibility, 41 studies were selected for inclusion in the systematic review (Figure 1). The studies included were published between 1993 and 2021 and were conducted in the following geographic areas: 4 in Asia (Korea), 6 in Australia, 9 in Europe (1 in Finland, 3 in Germany, 1 in Italy, 1 in Sweden, 1 in the United Kingdom, 1 in Austria, 1 in Portugal), and 22 in North America (4 in Canada, 18 in the United States). These 41 papers reported the results of 34 cross-sectional analyses and 7 longitudinal analyses (6 prospective and 1 retrospective cohort studies). Sample sizes ranged from 48 to 7,561.

### Interplay between LP and mental disorders

#### Lower LP predicting higher prevalence of mental disorders or symptoms severity

Thirty-five of the papers reviewed [22–25, 27–36, 38–40, 42,44,45, 47–52, 54–58, 60–63] found a significant association between low LP and mental disorders, with lower LP predicting higher prevalence of the disorder or worse severity of psychiatric symptoms. Of these papers, 13 studies focused only on the relation between depressive symptom severity and LP [22, 24–26, 28,32,36, 38–40, 42,47,61]; 4 studies investigated the correlation between LP and depression prevalence [35,51,55,56]; 2 focused on post-traumatic stress disorder (PTSD) symptom severity [58,62], 1 on PTSD prevalence [54], 1 on both PTSD and anxiety severity [31], 1 on psychosis prevalence [50], 1 on somatization prevalence [33], and 1 on somatic symptom severity [45]. Of the remaining 10 papers, four on the symptoms severity of more than one condition [44,49,52,63], whereas 7 focused on the prevalence of more than one mental disorder [23,27,29,30,34,48,57]. Finally, one study [57] retrospectively evaluated the charts of 243 migrants who needed to be hospitalized in a Psychiatry Unit between 2004 and 2018. Interestingly, subjects treated for their first-episode of any mental illness were found to be less proficient than those patients with a previous history of mental disorders.

#### Depression

Low LP was correlated with high prevalence of depression in five studies [30,34,35,51]. At the same time, depressive symptom severity assessed through different psychometric measures was found to correlate negatively with LP in 12 studies [22,26,28,32,36,38,40,42,52,55,61,64].

Among Afghan refugees resettled in Australia, participants presenting with more depressive symptoms reported higher levels of communication difficulties and lower LP compared to nondepressed subjects from the same community [55]. Asian and Korean immigrants with good LP showed a lower prevalence of lifetime psychiatric disorders and depression [30,34,35]. Similar results were found among women resettled in Portugal from different countries: the prevalence of postpartum depression observed in migrant women was higher than in native speakers, and the risk was higher for limited or intermediate proficiency groups compared to fully proficient migrant women [56]. Moreover, the effect of LP was stronger when the analysis was restricted to people with a recent history of migration [56]. Among Asians migrating to the United States or Canada, LP correlated with depressive symptom severity assessed by different measures [22,28,32,36,38,40,42]. In one case, the correlation was significant only among second-generation migrants [22] or among subjects with low ethnic identity [28]. Moreover, depression among first-generation migrants correlated with higher mother tongue usage (e.g., using only primary language at home) [22]. Among Korean migrants living in the United States, the correlation between English LP and depression severity assessed through the GDS-SF was found to be significant even after adjusting for sociodemographic factors, years in the United States, acculturative stress and self-reported discrimination [32,38,40]. Also, LP was a significant predictor for improvement of depressive symptom severity over time in asylum seekers living in Germany [52]. Among Latinos, higher LP correlated with lower CES-D scores [24,61], but this correlation was inverted in high ethnic density neighborhoods [61]. At the same time, LP was found to be a positive moderator of the detrimental effect of perceived discrimination on depression [24]. Also, gender differences impacted on the interplay between LP and mental health: LP did not affect the mental health of Asian women, when they were analyzed separately from men [30]. On the other hand, in a group of 200 women from F.S.U. living in the United States for fewer than 6 years, both LP and English usage had a significant inverse correlation with depressive symptom severity assessed by CES-D [26]. In the same sample, English usage but not LP explained 33.0% of CES-D score variance [26]. Interestingly, LP was positively correlated with employment status [22,51] and social interactions [22]. Furthermore, age, education, and LP at baseline predicted LP at follow-up [51]. Finally, gender differences impacted on language skills. In fact, LP was found to be lower in Asian female participants than in males at baseline, even if female participants’ skills improved over time [51].

#### Anxiety

Low LP was associated with higher prevalence of anxiety symptoms among migrants [29,48]. Among Russian and Kurdish women, LP significantly correlated with anxiety severity as assessed by the Hopkins symptoms checklist (HSCL-25) [44]. LP again significantly correlated with reduced anxiety among Vietnamese refugees resettled in the United States [31]. Interestingly, both gender and ethnicity differences impacted on anxiety symptom interplay with LP: Somali migrants and males did not show the same association found for Russian or Kurdish women [44]. Moreover, LP did significantly mediate the positive relationship between lifetime exposure to traumatic events and the severity of anxiety [48] and was a significant predictor for anxiety severity decrease over time in asylum seekers living in Germany [52].

#### Post-traumatic stress disorder

A negative association was shown between low LP and higher PTSD prevalence [48,65] or PTSD symptoms severity [52,62,63]. LP was a significant predictor of lower PTSD symptoms or their reduction over time among asylum seekers in Germany [52], Iraqi refugees [63], and Somali or Oromo young refugees living in the United States [62]. The same findings were replicated in Cambodian migrants, with greater LP being protective against PTSD [27]. Moreover, among Afghan refugees resettled in Australia, communication difficulties were more frequently reported by participants presenting with PTSD symptoms compared to non-PTSD subjects from the same community, with low LP predicting higher risk of PTSD in different multivariate models [54]. Interestingly, among Somali and Oromo refugees, LP varied significantly between men and women, with men reporting better language skills, but no gender differences were observed for mental disorder prevalence or severity [62]. Gender differences were highlighted also among humanitarian migrants in Australia with low LP (defined as higher linguistic barriers) predicting higher PTSD scores in male refugees but not in female ones [58]. Interestingly, even though they reported lower rates of PTSD, the effect of LP on PTSD seemed to be higher among male migrants arrived in Australia between 2016 and 2017 [58].

#### Somatic symptoms

Greater LP was associated with lower prevalence of somatization [33] and lower symptom severity [45], even after adjusting for demographic characteristics and migration factors.

#### Psychotic disorders

In a multicentric study conducted by Jongsma et al. on individuals with first-episode psychosis and healthy controls, including almost 35% of migrants in both groups, the authors defined “Linguistic Distance” combining LP in the host country language with a language-tree measured distance between first language and host country language [50]. The authors found an almost two-fold increase in the odds of psychosis among those reporting linguistic distance [50]. Moreover, linguistic distance was significantly associated with an ethnic minority background [50]. According to another study, individuals with psychosis presented greater odds of language-related problems than those diagnosed with major depressive disorder (MDD), bipolar disorder (BD), or substance use disorder (SUD) [23].

#### Subclinical symptoms and psychological distress

Four of the papers reviewed did not find any significant association between LP and clinically relevant major mental disorders; nevertheless, they found a negative relationship between LP and subclinical symptoms and/or psychological distress [25,39,47,49]. Low LP was associated with higher stress response evaluated by salivary cortisol levels [39], higher alienation [39], more pronounced “migration stress” [47] and lower health literacy [47]. Moreover, the exposure to premigratory trauma correlated with the severity of depression, and this relationship was mediated by LP: subjects with lower LP seemed to be more prone to developing depressive sequelae following exposure to premigratory trauma [25]. Among Puerto Ricans resettled in the United States because of Hurricane Maria, English language preference protected from psychological distress, as evaluated by the K-6 scale [49].

#### Better LP predicting higher prevalence of mental disorders or symptom severity

Two of the papers reviewed [46,66] found higher LP predicting higher prevalence [66] or severity [46] of psychiatric symptoms. Both papers assessed depression, PTSD, anxiety and other mental disorders in the same population [46,66]. Specifically, higher LP predicted a higher risk for both lifetime and past-year risk of psychiatric disorders (MDD, AD, and SUDs) among Latinos, with higher prevalence of MDD in males and AD in females [66]. Moreover, higher LP was related to higher anxiety symptom severity among female refugees resettled in Australia [46].

#### No relationship between LP and mental disorders

Four of the papers reviewed [37,41,43,53] did not find any significant association between LP and mental disorders. No significant relationships between LP and depressive symptoms severity were found among elderly Korean immigrants living in Canada [37] or female Asian immigrants residing in Korea after an international marriage [41]. No direct relationship was found between LP and depressive symptoms in Chinese mothers living with their children in the United States, even though LP was found to exert an indirect effect (mediated by economic and social factors) on depressive symptoms [53]. One other study did not find any significant relationships between LP and the prevalence of PTSD and grief-related symptoms [43].

### The influence of sociodemographic and migration factors on the relationship between LP and mental disorders

Among the studies collected, the evidence of a difference in mental disorder prevalence and/or severity between first- and second-generation migrants was insufficient. Seven studies included both first- and second-generation migrants [22,29,30,45,50,64,66], but only two of them assessed the correlation of LP and mental disorder prevalence and/or severity by migrant generation [22,50]. First-generation migrants showed stronger effects of language distance on risk of psychosis, whereas second-generation migrants seemed to be more affected by social disadvantages [50]. On the other hand, LP negatively correlated with the severity of both depression and psychological distress in a mixed sample of both first- and second-generation Chinese migrants, but, in adjusted models, the correlation between depression severity and LP was still significant only in the second-generation sample [22].

Four of the studies collected were based on mixed and balanced populations and found gender-related differences in the interplay between LP and mental disorders [30,39,44,66]. More specifically, among women but not men, higher LP was protective against alienation [39] and somatization [44]. On the other hand, among Asiatic migrants LP was a protective factor against lifetime and 12-month psychiatric disorder prevalence only among male participants [30]. Among Latinos, highly proficient men showed a higher risk of lifetime MDD, while a higher risk of lifetime AD was found for more proficient women [66]. Both men and women with better LP showed an increased risk of SUD [66]. Eight more studies were based on women-only samples [26,29,36,41,42,46,53,56]: one of them found a negative correlation between LP and AD prevalence rates [29]; one of them found that LP was negatively related with postpartum depression prevalence [56]; two of them found that LP negatively correlated with depression severity [26,36]; three studies did not find any correlations between LP and depression severity [41,42,53]; and one found that greater LP predicted more pronounced anxiety symptoms [46]. Two more studies were unbalanced, with more than 75% female participants [47,61]. In the only study based on a sample of males alone, depressive symptom severity was found to correlate negatively with LP [22].

### Quality assessment and limitations

The QATSO Score for each study is reported in Table 1. The most frequent limitations were the lack of validated instruments for LP assessment and the absence of information about response rate among surveyed individuals in most studies. Furthermore, migration history data are missing in 12 papers, while about 15 did not provide information about privacy or ethical requirements or failed to meet them. Sampling methods representative of the population were employed in most studies, as well as validated psychiatric instruments and checking for confounding factors.

## Discussion

Our work of review shows that the large majority of the studies reported a significant association between lower LP and higher prevalence and/or severity of psychiatric symptoms and mental disorder. Only two records found the opposite relationship while four papers reported no association. Low LP was consistently associated with several mental illnesses in migrants, including psychosis, mood, anxiety, and PTSDs. This result was replicated in both cross-sectional and longitudinal studies. Most of the studies included had a cross-sectional design, which do not allow to draw causal relationships. Notably, all the longitudinal studies included in this review reported a positive effect of LP acquisition over time on the prevalence of mental disorders [51] or symptom severity [52,61,63], suggesting a protective effect of LP on mental health.

As is known, most studies showed that migrants present higher risk of severe mental disorders than natives. Several hypotheses have sought to explain the poor mental health in migrants. Both pre- and postmigratory factors such as lack of social support, social networks and social opportunities in the host country have been invoked [4,5,67], and LP may play a role in this process [50]. Deficits in social skills and executive functions have proved to be a key feature of several mental disorders and may prevent appropriate LP acquisition, exacerbate isolation and lead to psychiatric symptoms [68]. In this light, poor LP may be considered as a phenotype of the vulnerability to psychiatric disorders. At the same time, low LP may impair social functioning in the host country, which is well known to be related to poor mental health. On the contrary, the results of this review suggest that adequate LP might promote migrants’ inclusion in all the main societal areas and thus positively shape migrants’ adaptation and mental health in both the short- and the long-term.

Interestingly, some findings support the hypothesis that LP may have differing degrees of relevance through the postmigratory phase. Upon arrival in the host country, LP acquisition has been shown to be associated with cultural conflict and a sense of alienation. In this early postmigration phase, a strong ethnic identity along with adequate social support may be helpful for adaptation and psychological wellbeing of migrants. During later stages of the postmigration phase, however, an adequate LP and familiarity with the host culture are decisive for both migration and mental health outcomes. For instance, higher depression prevalence rates have been found among migrants who reported insufficient LP about 10 years after the initial migration [51]. Again, there is evidence that good LP is associated with lower levels of postmigratory social disadvantage: good LP has been linked with better education [37,42,51,60], more affective relationships with the host population [22], nonsingle marital status [37], having health insurance [69], and higher levels of postmigration occupation [22,28,51].

With regard to generation differences, our review shows that findings on the association between LP and poor mental health vary in relation to specific disorders: while less proficient first-generation migrants seemed more vulnerable to psychosis [50], second generations were more prone to depression [47]. Further studies should clarify these associations in order to achieve better understanding of the etiopathogenic mechanisms underlying the differential effect of LP on mental health by generational status.

Migrant women are often considered more vulnerable to psychiatric disorders [70,71]. We found insufficient evidence of gender-related differences in the interplay between LP and mental disorders prevalence and/or severity. Interestingly, two studies found a difference in LP between male and female participants [28,62], with women showing lower LP at the time of migration but significantly greater improvement over time.

### Strengths and limitations

The strength of our study is the global prospective based on the extensive literature review carried out including studies conducted in a variety of countries and over a 30-year timespan. Despite this strength, we have to recognize that the majority of studies included present several limitations: (a) the lack of validated LP assessment tools resulting in high heterogeneity in LP assessment within studies, potentially contributing to differences in LP estimates; (b) the heterogeneity in psychopathological assessment instruments (Table S2) which may have resulted in different estimates of mental disorder prevalence and severity across the review; (c) the cross-sectional design of most of the studies included, which does not allow for causal relationships; (d) the heterogeneity of study samples in age, gender, education level, and ethnicity, which may reflect differences in target populations as well as in sampling design. Furthermore, only a few studies investigated the link between LP and psychosis [16,39]. The lack of sample, outcome, and methods homogeneity among the reviewed studies may be responsible for the different estimates of the interplay between LP and mental health. Such heterogeneity has not allowed to conduct meta-analysis and might limit the generalizability of results. Finally, low LP may be considered as one of the main barriers involved in reducing migrants’ access to health services and thus limiting the participation in the studies [22]: the samples included may thus not be completely representative of the general population of migrants with mental disorders.

## Conclusions

Low LP was generally found to negatively affect migrants’ mental health, being associated with increased prevalence and severity of psychiatric symptoms as well as of mental disorders. This finding seems to indicate that people who are not proficient in the majority language may encounter mental health issues and consequent poor social and job functioning. In the light of our results, we would expect that early and effective interventions targeting migrants’ LP could reduce both incidence and prevalence of mental disorders among migrants in the medium-long term. Given these evidences, clinicians treating migrants with mental disorders should consider integrating interventions aimed at supporting language literacy as part of their individualized care programs.

European countries requiring LP in order to obtain permanent residence increased from one in 1990 to 18 in 2014 [72]. In some countries, language acquisition is supported by language courses and, in some cases, language or integration courses are mandatory for migrants and asylum seekers during the process of nationality acquisition [72]. Several no-profit organizations provide free language classes for migrants in different countries. Anyway, only two countries worldwide actually provide freely available language courses for migrants upon arrival (Sweden and Canada) [72]. Recently, Morrice and colleagues published an interesting article entitled “You cannot have a good integration when you do not have a good communication” [73]. We would like to readopt this concept and say that “you cannot have good mental health if you do not have a good communication.” We strongly hope that the present findings will inspire authorities to provide support and courses to improve migrants’ LP upon arrival. At the same time, in order better to evaluate the relationship between LP and psychiatric disorders we need longitudinal studies on larger samples of migrants.
